# Treatment of severe and moderate acute malnutrition in low- and middle-income settings: a systematic review, meta-analysis and Delphi process

**DOI:** 10.1186/1471-2458-13-S3-S23

**Published:** 2013-09-17

**Authors:** Lindsey M Lenters, Kerri Wazny, Patrick Webb, Tahmeed Ahmed, Zulfiqar A Bhutta

**Affiliations:** 1 Centre for Global Child Health, The Hospital for Sick Children, Toronto, ON, Canada; 2 Friedman School of Nutrition Science and Policy, Tufts University, Boston, MA, USA; 3 Centre for Nutrition and Food Security, ICDDR,B, Dhaka, Bangladesh; 4 Division of Woman and Child Health, Aga Khan University, Karachi, Sindh, Pakistan

## Abstract

**Background:**

Globally, moderate acute malnutrition (MAM) and severe acute malnutrition (SAM) affect approximately 52 million children under five. This systematic review evaluates the effectiveness of interventions for SAM including the World Health Organization (WHO) protocol for inpatient management and community-based management with ready-to-use-therapeutic food (RUTF), as well as interventions for MAM in children under five years in low- and middle-income countries.

**Methods:**

We systematically searched the literature and included 14 studies in the meta-analysis. Study quality was assessed using CHERG adaptation of GRADE criteria. A Delphi process was undertaken to complement the systematic review in estimating case fatality and recovery rates that were necessary for modelling in the Lives Saved Tool (LiST).

**Results:**

Case fatality rates for inpatient treatment of SAM using the WHO protocol ranged from 3.4% to 35%. For community-based treatment of SAM, children given RUTF were 51% more likely to achieve nutritional recovery than the standard care group. For the treatment of MAM, children in the RUSF group were significantly more likely to recover and less likely to be non-responders than in the CSB group. In both meta-analyses, weight gain in the intervention group was higher, and although statistically significant, these differences were small. Overall limitations in our analysis include considerable heterogeneity in many outcomes and an inability to evaluate intervention effects separate from commodity effect. The Delphi process indicated that adherence to standardized protocols for the treatment of SAM and MAM should have a marked positive impact on mortality and recovery rates; yet, true consensus was not achieved.

**Conclusions:**

Gaps in our ability to estimate effectiveness of overall treatment approaches for SAM and MAM persist. In addition to further impact studies conducted in a wider range of settings, more high quality program evaluations need to be conducted and the results disseminated.

## Introduction

Globally, approximately 33 million children under five years of age are affected by moderate acute malnutrition (MAM), defined as a weight-for-height z-score (WHZ) between -2 and -3, and at least 19 million children under five by severe acute malnutrition (SAM), defined as a WHZ of <-3 [1,2]. For children with SAM, the risk of death is approximately 10-fold higher compared to children with a z-score ≥ – 1 [3]. Based on an analysis by UNICEF, WHO and the World Bank [2], 32 of 134 countries for which there was data on prevalence of acute malnutrition (WHZ <-2) had a prevalence of 10% or more – a threshold that represents a “public health emergency requiring immediate intervention” [2]. This analysis also showed that, since 1990, prevalence rates of wasting (acute malnutrition, WHZ <-2) have declined three times more slowly than for stunting (chronic malnutrition, height-for-age z-score <-2), decreasing by 11% and 35% respectively.

SAM inpatient management guidelines have been published by the World Health Organization (WHO), and updates to the protocol are pending [1,4]. Practitioners and WHO experts endorse community-based management for uncomplicated SAM while still advising that children who are severely malnourished and have medical complications, such as severe oedema, should be treated in an appropriate health facility [1,5]. With respect to the management of MAM, there are several published guiding documents [6-8] and there is ongoing interaction among researchers, practitioners and policy makers; however, there is currently no standardized approach to the management of MAM.

Since the early 2000s, the products used to deliver nutrients for management of SAM and MAM and the approaches used to target and deliver these products have been evolving rapidly. Innovations include new formulations and packaging and a shift from institutional to community-based management. Specially formulated foods are the cornerstone of treatment programs and include fortified blended foods (e.g. corn-soy blend (CSB)) as well as ready-to-use-foods (RUFs). RUFs are nutrient-dense products that are formulated as lipid pastes, bars or biscuits that provide specified amounts high quality of protein, energy and micronutrients, depending on the target population [9]. Detailed summaries of RUF types have been described elsewhere [7].

Specific aspects of inpatient management of SAM, for example approaches to treating infectious, IV fluid for shock, management of diarrhea in SAM and management of micronutrient deficiencies [10] as well as antibiotic use in SAM management [11,12] have been reviewed. Nonetheless, there has not been a systematic review of the WHO protocol in its entirety, compared to standard care. This is essential for understanding whether the protocol is effective as a package. In 2008, a preliminary review of approaches to treat SAM was undertaken for the Lancet Maternal and Child Undernutrition Series [13]; however, this review included more broadly defined cases of undernutrition, was not specific to children under five years, and included trials of variable quality and methods.

Two recently published Cochrane reviews have also investigated specially formulated foods for treating children with MAM [14] and SAM [15] and RUTF for home-based treatment of SAM in children 6 months to 5 years of age [15], and while the details of the analyses vary somewhat, the overall conclusions are congruous with the analyses presented here. Other reviews of community-based management of SAM as well as management of MAM have been conducted [16-18]; however, these reviews typically did not include meta-analyses and included studies using definitions of malnutrition, such as weight-for-age, which are not all specific to acute malnutrition.

We undertook a systematic review in order to evaluate the effectiveness of approaches to managing SAM, including the WHO protocol for inpatient management [4] and community-based management using RUTF [5], as well as the effectiveness of approaches to managing MAM. Our review focused on children under five in low- and middle-income countries. In addition, we aimed to identify gaps in the literature and to generate the effect estimates necessary for including these interventions in the Lives Saved Tool (LiST). LiST models the reduction in child deaths by specific causes associated with increasing coverage of individual interventions. Recent mortality rates and cause of death data for newborns, infants, and children are incorporated, by country, using estimates established by the Child Health Epidemiology Group (CHERG) [19].

## Methods

### Searches

We developed comprehensive search strategies for the following databases: Medline, Embase, Web of Science, WHO regional databases and the Cochrane library (see additional file 1). We conducted hand searches for sources of grey literature, including the Emergency Nutrition Network and Epicentre websites, Grey Literature Review and the World Bank website. We also issued a call to key non-governmental organizations requesting reports from their programs.

Literature published after 1970 was included and we did not restrict by language. Searches were conducted between October 9, 2012 and November 3, 2012. We did not limit by study design type or by publication type when selecting studies for inclusion. However, we excluded before-and-after studies in the meta-analysis, as we were not confident in the abilities of these studies to isolate the intervention effect separately from the confounding variables.

We defined MAM as weight-for-height z-score (WHZ) between -2 and -3 standard deviations (SD), weight-for-height (WFH) 70-80% of the NCHS or WHO reference median or mid-upper arm circumference (MUAC) of 115-125mm. We defined SAM as weight-for-height z-score (WHZ) <-3 SD, weight-for-height (WFH) <70% of the median NCHS or WHO reference or mid-upper arm circumference (MUAC) <115mm or oedema [4].

We contacted authors who used alternative definitions of acute malnutrition or in cases where there was insufficient information available in the publications to request additional information or disaggregated data. An asterisk next to the authors’ names in the forest plots indicates the use of unpublished data.

### Data synthesis and quality assessment

We coded and categorized the types of interventions in each article. For moderate acute malnutrition, we conducted a meta-analysis only on ready-to-use-supplementary food (RUSF) compared with CSB, as this was the only comparison with multiple studies that could be pooled. Likewise, for severe acute malnutrition, we conducted a meta-analysis on RUTF compared with standard therapy. No study has included true control groups using placebo or no intervention for ethical reasons. We also conducted a meta-analysis on two studies comparing inpatient treatment to ambulatory treatment for children with SAM and MAM, as well as a meta-analysis comparing locally-produced RUTF to imported RUTF for rehabilitation of children with SAM.

We included outcomes needed for LiST and those routinely used for program monitoring. These include: mortality, recovery rate (as defined by authors), relapse rate (as defined by authors), default rate, time to recovery, and change in anthropometric measures such as weight, height, MUAC and WHZ. Outcomes with more than one data point were included in the final analysis.

We used a standardized data abstraction form to collect information regarding study characteristics, descriptions of the interventions and comparisons, outcomes of interest and effects as well as quality of the studies. We assessed quality based on the CHERG adaptation of the GRADE technique at the individual study level and at the outcome level [20]. Studies were classified as high, moderate, low or very low quality. The quality of each study was assessed based on study design, methods and generalizability.

Quality assessment at the outcome level was graded based on volume and consistency of the evidence, strength of the pooled effect and strength of statistical evidence based on the p-value. Levels of heterogeneity were assessed by visual inspection, looking for overlapping confidence intervals, and by the I^2^ value. An I^2^ value of >50% was considered to be evidence of substantial heterogeneity.

The meta-analysis was conducted using RevMan 5.2®. We applied generic inverse variance methods to all analyses and used a random effects model in all cases; summary estimates are presented as relative risk (RR) or mean difference (MD) and 95% confidence intervals (CI).

## Results

**Figure 1:**
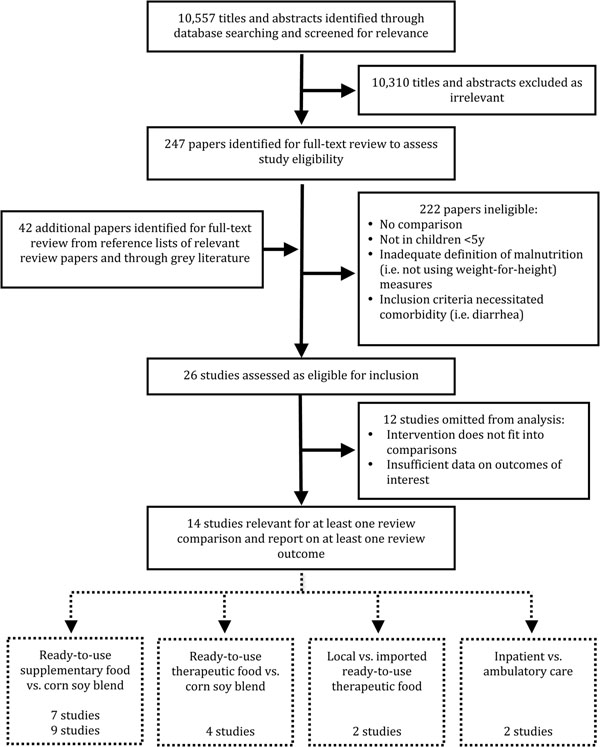
Flow diagram showing identification of studies included in the review

Our search identified 10,557 titles. Screening of these titles, full text review and data abstraction was done independently by two team members and then matched. Any disagreements were resolved through discussion or, where necessary, through consultation with a third team member. After screening titles and abstracts retrieved by our search, 10 310 articles were excluded as clearly unrelated. The full-texts of 247 papers were screened and 26 papers were identified to meet the inclusion criteria. Twelve studies were subsequently omitted from the meta-analysis as we could not pool their interventions and/or there was insufficient data on any outcomes of interest. A total of 14 studies were included in the final meta-analysis (see figure 1).

The results have been categorized by intervention type, and whether severe or moderate acute malnutrition was addressed. All of the trials were situated in areas of protracted food insecurity where wasting is endemic. While prevalence of wasting will certainly respond to fluctuating environmental factors, none of the studies represented a situation of sudden and acute starvation. Forest plots for mortality, recovery rate and weight gain are presented in the text; however, all forest plots can be found in additional file 2.

### Community-based management of severe acute malnutrition: Therapeutic feeding with RUTF vs. standard therapy

Three articles, representing two unique trials, were located that compared community-based management with RUTF versus standard therapy in children with severe acute malnutrition [21-23]. Standard therapy entailed treatment in an inpatient facility until complications resolved, with the subsequent provision of Corn-Soy blend (CSB) for feeding the child at home. Both were quasi-experimental trials set in Malawi. In one trial, all children were treated for infectious and metabolic complications in a nutritional rehabilitation unit; they were enrolled into the trial upon discharge and given either RUTF or CSB for home-treatment [21,23]. The other trial enrolled children upon discharge from a nutritional rehabilitation unit as well as directly from the community. All of the children in the standard therapy group and about half of the children in the RUTF group had received inpatient treatment [22]. The first did not test children for HIV, but presumably included a mix of children who were HIV-infected and HIV-uninfected and took place from 2002 to 2003 [22]. The other two articles reported data from the same trial, which took place in 2001. One reported data on the HIV-infected cohort [23], while the other reported data for children who were HIV-uninfected [21]. We assessed the quality of the studies as moderate/low [22] and moderate/high [21,23].

**Figure 2:**
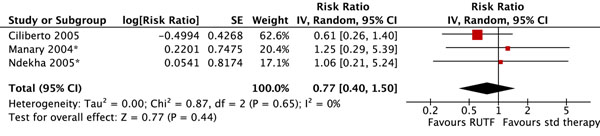
Forest plot for the effect of RUTF vs. standard (std) therapy on mortality in SAM

**Figure 3:**
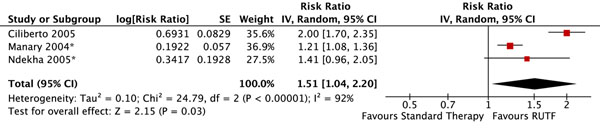
Forest plot for the effect of RUTF vs. standard therapy on recovery in SAM

**Figure 4:**
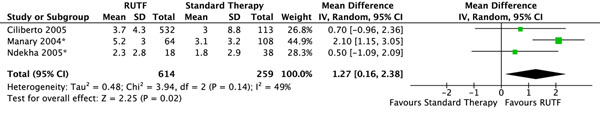
Forest plot for the effect of RUTF vs. standard therapy on weight gain in SAM

There were no significant differences in mortality (figure 2). Children who received RUTF were 1.51 times more likely to recover (defined as attaining WHZ ≥ -2) than those receiving standard therapy (RR: 1.51, 95% 1.04 to 2.20) (figure 3). There was substantial heterogeneity (I^2^ = 92%), the effect was only marginally statistically significant, and this outcome was graded as low quality (see table 1 for quality assessment). Children who received RUTF had a greater average height gain (MD: 0.14, 95% CI 0.05 to 0.22) and MUAC gain (MD: 0.11, 95% CI 0.05 to 0.18); both outcomes were graded as moderate/low quality. Average weight gain in the RUTF group was also greater (MD: 1.27, 95% CI 0.16 to 2.38) and this outcome was graded as moderate quality (figure 4).

**Table 1:** Severe acute malnutrition: quality assessment of evidence at the category level

	QUALITY ASSESSMENT				SUMMARY OF FINDINGS		
**Number of studies**	**Design**	**Limitations**	**Consistency**	**Generalizability to population of interest**	**Events in intervention group**	**Events in control group**	**Effect size (95% CI)**

**Mortality: Moderate outcome specific quality**					**Risk Ratio**		

3	quasi-experimental	1 moderate/low, 2 moderate/high quality studies	Inconsistent direction of effect, I^2^ = 0	Children 10-60 months presenting to nutrition rehabilitation units in Malawi	25	15	0.77 (0.40, 1.50)

Recovery rate: Low outcome specific quality					**Risk Ratio**		

3	quasi-experimental	1 moderate/low, 2 moderate/high quality studies	Consistent direction of effect to varying degree, I^2^ = 92%	Children 10-60 months presenting to nutrition rehabilitation units in Malawi	148	155	1.51 (1.04, 2.20)

Rate of height gain: Moderate/low outcome specific quality					**Mean Difference**		

3	quasi-experimental	1 moderate/low, 2 moderate/high quality studies	Inconsistent direction of effect, I^2^ = 40%	Children 10-60 months presenting to nutrition rehabilitation units in Malawi	N/A	N/A	0.14 (0.05, 0.22)

Rate of MUAC gain: Moderate/low outcome specific quality					**Mean Difference**		

3	quasi-experimental	1 moderate/low, 2 moderate/high quality studies	Inconsistent direction of effect, I^2^ = 0	Children 10-60 months presenting to nutrition rehabilitation units in Malawi	N/A	N/A	0.11 (0.05, 0.18)

Rate of weight gain (RUTF vs. standard care): Moderate outcome specific quality					**Mean Difference**		

3	quasi-experimental	1 moderate/low, 2 moderate/high quality studies	Consistent direction of effect to varying degree, I^2^ = 49%	Children 10-60 months presenting to nutrition rehabilitation units in Malawi	N/A	N/A	1.27 (0.16, 2.38)

Rate of weight gain (imported vs. locally-produced RUTF): Moderate/low outcome specific quality					**Mean Difference**		

2	quasi-experimental, RCT	1 moderate/high and 1 low quality study	Consistent direction of effect, I^2^ = 0%	Children 6-60 months presenting to feeding clinics, studies in Malawi and Senegal	N/A	N/A	0.53 (-0.57 to 1.63)

### Facility-based management of severe acute malnutrition: WHO protocol for inpatient management of SAM vs. standard care

A literature review by Schofield and Ashworth [24] indicates that between the 1950s and 1990s, case fatality rates (CFR) were typically 20-30% among children treated for SAM in hospitals or rehabilitation units. Average CFR was higher (50-60%) among children with oedematous malnutrition. The persistence of high CFR was attributed to faulty case management, and the authors called for prescriptive treatment guidelines as part of a comprehensive training program. In the 2008 Lancet Maternal and Child Undernutrition Series, the preliminary review estimated that treating children according to the WHO Protocol compared to standard care would result in a 48% reduction in deaths (RR 0.52, 95% CI 0.43, 0.64) [13]. This was used in the model to determine impact on mortality for SAM but requires refinement for the reasons mentioned in the introduction.

In terms of recent studies, we found one before/after study [25], two retrospective chart reviews [26,27], one quasi-experimental study [28] and four prospective cohorts [29-33] that examined the case fatality rates and recovery rates of children with SAM treated according to the WHO protocol or an adapted WHO protocol. There was also one cluster RCT that compared inpatient treatment to home-care and day-care treatment [34,35]; this study contained methodological issues and did not adequately describe the intervention (see additional file 3 for study assessment).

None of the studies provided sufficient information to ensure that each step of the WHO protocol was followed and many noted variations from the protocol. One study [31,32] excluded children with severe complications and thus may not be generalizable. Case fatality rates ranged from 3.4% to 35% (see table 2). The highest CFR stemmed from a cohort of HIV-infected children [31,32], while the lowest rate was achieved in a study that provided few details on the protocol followed [34]. Only two studies provided information on recovery rates, which were 79.7% and 83.3%, respectively [28,31,32].

**Table 2:** Characteristics of studies reporting case fatality for inpatient treatment of SAM according to WHO protocol

Study	Country	Study Design	Intervention	Variance from WHO protocol and study design issues	CFR for inpatient group
Bachou 2008	Uganda	Before and after	Improved practice to reduce unnecessary blood transfusions and IV infusions was in accordance with the WHO guidelines	Micronutrients and parenteral antibiotics given in accordance with Ministry of Health recommendations; measles vaccine and sensory stimulation not mentioned	25%
Berti 2008	Ethiopia	Retrospective cohort	Treated according to adapted UNICEF (2004) guidelines	Not clear if micronutrient supplementation aligns with WHO protocol; sensory stimulation not mentioned	7%
Chinkhumba 2008, Fergusson 2009	Malawi	Prospective cohort	Nutritional rehabilitation in accordance with Malawi Ministry of Health guidelines (2003), adapted from WHO guidelines (2003)	HIV-infected children not given ART; unclear approach to rehydration, provision of micronutrients, antibiotics and sensory stimulation; children with severe complications not included	HIV-infected: 35% HIV-uninfected: 10%
Hossain 2009	Bangladesh	Quasi-experimental	Treated according to WHO protocol	Protocol not described	7%
Manary 2000	Malawi	Prospective cohort	Treated according to 1971 WHO standards	Children fed at slower rate; did not use ReSoMal ORS; included an additional intensive nursing component; measles vaccination not mentioned	25%
Maitland 2006	Kenya	Retrospective cohort	Treated according to WHO guidelines insofar as staffing allowed	Fed at a higher rate initially; authors state that WHO protocol used but not described in detail.	19%
Ahmed 1999	Bangladesh	Prospective cohort	Adapted WHO criteria	Children fed at slower rate; all children had diarrhea; acute malnutrition assessed using either WFH or WFA	9%
Khanum 1994 and 1998	Bangladesh	cRCT	Protocol not described	High risk of bias with respect to randomization; carers often requested to change groups	3.40%
Ashworth 2004	South Africa	Prospective cohort	Managed according to WHO guidelines insofar as staffing permitted	Age range of children not given; unclear if all children have SAM as defined by WHO	24%

WHO: World Health Organization

UNICEF: United Nations Children’s Fund

HIV: Human immunodeficiency virus

cRCT: Cluster randomized controlled trial

WFH: weight-for-height

WFA: weight-for-age

ORS: oral rehydration solution

Ashworth noted that issues of training were paramount to improving outcomes, as mortality rates increased with the influx of new, untrained doctors into a hospital [29]. Two additional observational studies documented that implementing changes to dietary and clinical management did not seem to be sufficient to promote substantial reductions in case fatality rates. Key factors associated with improved outcomes were related to quality of care and institutional culture, including staff morale, attentiveness of nurses and support structures at the managerial level [36,37].

### Community-based management of moderate acute malnutrition: Supplementary feeding with RUSF vs. CSB

Our review identified five studies investigating the effect of Ready-to-Use Supplementary Food (RUSF) compared to Corn Soy Blend (CSB) in moderately malnourished children under five years of age [38-42]. Two of the studies were cluster randomized controlled trials (cRCTs), one set in 10 health centres and health posts in the Sidama zone of Ethiopia [39] and the other in the Dioila health district in Mali [38]. Three of the studies were randomized controlled trials (RCTs). Two were located in southern Malawi [40,41], and one in the Zinder region of southern Niger [42]. Two studies took place from 2007 to 2008 [38,42]; the remaining three studies took place during 2009 and 2010 [39-41]. We assessed the quality of the studies to be low [42], moderate [38], moderate/high [39,40] and high [41] quality (see additional file 3).

**Figure 5:**
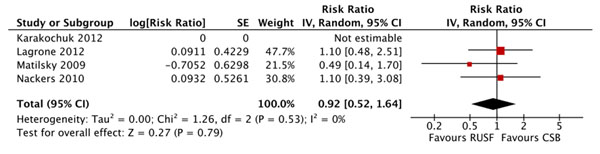
Forest plot for the effect of RUSF vs. CSB on mortality in MAM

**Figure 6:**
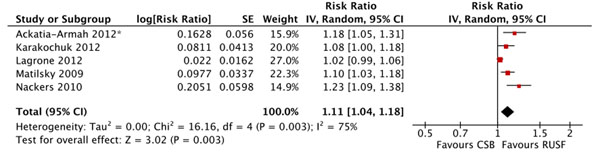
Forest plot for the effect of RUSF vs. CSB on recovery in MAM

**Figure 7:**
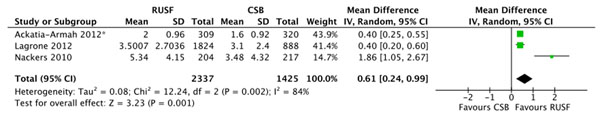
Forest plot for the effect of RUSF vs. CSB on weight gain in MAM

There were no significant differences in mortality for children given RUSF compared to those who received CSB (figure 5). However, the non-response rate was significantly lower in the RUSF group (RR: 0.65, 95% CI 0.47 to 0.90). This outcome has considerable heterogeneity (I^2^ = 57%) and was graded as moderate quality (see table 3). Children in the RUSF group were also significantly more likely to recover (RR: 1.11, 95% CI 1.04 to 1.18), although this estimate contained substantial heterogeneity and was graded as moderate/low quality (figure 6). The rate of height gain did not differ between the intervention and comparison groups. Children who received RUSF had an average MUAC gain of 0.04 mm per day (MD: 0.04, 95% CI 0.01 to 0.07) and had an average weight gain of 0.61 g/kg/d higher (MD: 0.61, 95% CI 0.24 to 0.99) than those who received CSB (figure 7). Upon discharge or completion of the study, children who received RUSF had an average weight-for-height z-score that was 0.11 z-scores greater than those who received CSB (MD: 0.11, 95% CI 0.04 to 0.17). While this is statistically significant, it may not be a sufficient difference to be clinically important. Nackers et al. [42] followed-up with children 6 months post-intervention. There were no significant differences in sustained recovery (defined as maintaining WFH ≥ 80% of the NCHS median post-treatment) or height gain.

**Table 3:** Moderate acute malnutrition: quality assessment of evidence at the category level

	Quality Assessment				Summary of Findings		
**Number of studies**	**Design**	**Limitations**	**Consistency**	**Generalizability to population of interest**	**Events in intervention group**	**Events in control group**	**Effect size (95% CI)**

**Mortality: Moderate/high outcome specific quality**					**Risk Ratio**		

4	RCT/ cRCT	1 high, 2 moderate/high and 1 low quality study	1 of 3 studies shows beneficial effect, I^2^=0%	Children 6-60 months presenting to health centres with MAM, all in Africa	30	20	0.92 (0.52 to 1.64)

**Non-response rate: Moderate outcome specific quality**					**Risk Ratio**		

4	RCT/ cRCT	1 high, 2 moderate/high and 1 low quality study	All studies show beneficial effect, I^2^ = 57%	Children 6-60 months presenting to health centres with MAM, all in Africa	176	312	0.65 (0.47 to 0.90)

**Recovery rate: Moderate/low outcome specific quality**					**Risk Ratio**		

**5**	RCT/ cRCT	1 high, 2 moderate/high, 1 moderate & 1 low quality study	All studies show beneficial effect, heterogeneous (I^2^ = 75%)	Children 6-60 months presenting to health centres with MAM, all in Africa	2,992	1,918	1.11 (1.04 to 1.18)

**Rate of height gain: Moderate outcome specific quality**					**Mean Difference**		

**2**	RCT	1 moderate/high, 1 low quality study	Consistent, I^2^ = 0%	Children 6-60 months presenting to health centres with MAM, all in Africa	N/A	N/A	-0.00 (-0.02 to 0.02)

**Rate of MUAC gain: Moderate outcome specific quality**					**Mean Difference**		

**2**	RCT	1 moderate/high, 1 low quality study	Consistent, I^2^ = 0%	Children 6-60 months presenting to health centres with MAM, all in Africa	N/A	N/A	0.04 (0.01 to 0.07)

**Rate of weight gain: Moderate/low outcome specific quality**					**Mean Difference**		

**3**	RCT	1 moderate/high, 1 moderate and 1 low quality study	Heterogeneous (I^2^ = 84%)	Children 6-60 months presenting to feeding centers with MAM, all in Africa	N/A	N/A	0.61 (0.24 to 0.99)

**Weight-for-height z-score change at completion or discharge: Moderate/low outcome specific quality**					**Mean Difference**		

**2**	RCT	1 high, 1 low quality study	Heterogeneous (I^2^ = 46%)	Children 6-60 months presenting to feeding centres with MAM, all in Africa	N/A	N/A	0.11 (0.04 to 0.17)

(c)RCT: (cluster-) randomized controlled trial

The comparison groups in two of the studies used standard CSB [39,41]. Two of the studies used “CSB++”, which contains a revised micronutrient profile and contains higher quality protein through the addition of milk powder [38,40] and the last study used “CSB-based pre-mix”, which contains additional oil and sugar [42]. We performed a sensitivity analysis, separating out studies using improved CSB (CSB++ and CSB-based pre-mix) from those using standard CSB. For mortality, the two studies using improved-CSB slightly favoured the comparison group, while the study using standard CSB favoured the intervention group. For the rate of height gain, there is a very slight difference in the direction of effect, but again there was no significant difference between the subgroups. The remaining outcomes showed consistent directions of effect. There were no significant differences between the subgroups for any outcomes.

### Severe acute malnutrition: Local vs. imported RUTF

**Figure 8:**
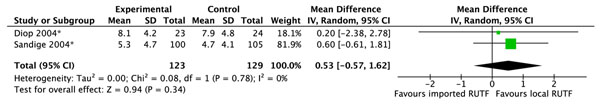
Forest plot for the effect of local vs. imported RUTF on weight gain in SAM

Two trials, one in Senegal, graded as low quality [43] and the other in Malawi, graded as moderate quality [44], compared imported RUTF to locally produced RUTF used in community-based management of SAM. There was no significant difference in weight gain between intervention groups (figure 8). This effect was consistent (I^2^ = 0%) and the overall outcome was graded as moderate/low quality.

### Severe and moderate acute malnutrition: Inpatient vs. ambulatory care

Two studies compared home-based treatment to inpatient treatment in children without severe complications. One moderate quality study in Niamey, Niger, enrolled children with MAM and SAM who were about to be discharged from the hospital [45]. The other study, graded as low quality, allocated children presenting to the nutrition unit in Dhaka, Bangladesh, to receive either home-based or inpatient care [35]. A third arm of this trial, day care, was not analyzed because it could not be pooled.

**Figure 9:**
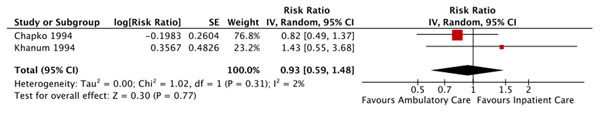
Forest plot for the effect of impatient vs. ambulatory care on mortality in SAM

There was no significant difference in mortality between home-based or inpatient care (figure 9). However, the studies produced opposite directions of effect and the overall quality of this outcome was rated as very low due to issues with study design, analysis and availability of key study details (table 4).

**Table 4:** Moderate and severe acute malnutrition: quality assessment of evidence at the category level

	Quality Assessment				Summary of Findings		
**Number of studies**	**Design**	**Limitations**	**Consistency**	**Generalizability to population of interest**	**Events in intervention group**	**Events in control group**	**Effect size (95% CI)**

**Mortality: Very low outcome specific quality**					**Risk Ratio**		

2	RCT	1 moderate and 1 low quality study	Inconsistent direction of effect, I^2^ = 2%	Children 5-60 months admitted to nutrition unit of hospitals in Bangladesh and Niger	26	29	0.93 (0.59 to 1.48)

### Results from additional studies not included in meta-analysis

We identified several interesting singular studies that could not be pooled in the meta-analysis. Oakley et al. [46] studied the effect of an RUTF consisting of 25% milk versus another with 10% milk in treating children with SAM, and found that the RUTF with the higher milk content was associated with a statistically significant higher recovery rate (p<0.05). A trial in urban Senegal randomized children with SAM and MAM to receive RUTF or F100 (a therapeutic milk-based product used for nutritional rehabilitation in inpatient facilities) [47]. The study found a statistically significant difference in the rate of weight gain: children who received RUTF gained an average of 5.50 g/kg/d more than those receiving F100 (MD: 5.50, 95% CI 3.00 to 8.00). Branger et al. [48] investigated the effect of adding spirulina, a microscopic algae to standard treatment or standard treatment plus fish for children with moderate and severe malnutrition in Burkina Faso. The authors found no significant differences between treatment groups.

Navarro-Colorado [49] found no significant differences in duration of rehabilitation or weight gain in children with severe acute malnutrition randomized to receive F100 or BP100, a ready-to-use food in biscuit form, although children receiving BP100 had a significantly higher energy intake. Finally, a double-blind, randomized, placebo controlled efficacy trial investigated the effect of adding probiotics and prebiotics to RUTF compared to standard RUTF. The study found no significant difference in the primary outcome, nutritional recovery, or any of the secondary outcomes, including mortality. HIV-infected children were at a higher risk of death in both groups, but HIV did not confound or modify the non-statistically significant effect of the intervention [50].

We found very few rigorous trials that compared the provision of therapeutic or supplementary foods to other types of interventions aimed at modifying the upstream factors that contribute to the development of wasting. Fauveau [51] compared education on appropriate complimentary feeding to education plus supplementary feeding in children aged 6-12 months. The supplementary food package contained rice, wheat, lentil power and cooking oil. While the group receiving the supplemental feeding package had a significantly higher monthly weight gain at three months, this result was not sustained at six months of follow-up.

A new randomized controlled trial comparing RUTF to RUTF plus antibiotics (either amoxicillin or cefdinir) in children with uncomplicated SAM in an outpatient setting found that the mortality rate was significantly higher in children receiving placebo than either antibiotic arm (amoxicillin RR: 1.55, 95% CI 1.07-2.24; cefdinir RR: 1.80, 95% CI 1.22-2.64) [52]. The proportion of children who recovered was significantly lower among the placebo arm compared to either antibiotic arm (amoxicillin: 3.6%, 95% CI 0.6-6.7; cefdinir: 5.8% lower, 95% CI 2.8-8.7), with no significant differences in death or recovery between the two antibiotic arms. Rates of weight gain among children who recovered were higher in the antibiotic arms compared to the placebo arm. HIV status was not known for over half of the children in the study. Additional studies are needed to strengthen the evidence base on whether children with uncomplicated SAM should be provided routine antibiotics.

## The Delphi process for establishing expert consensus

Our review found limited high quality comparative trials evaluating the package of care offered through community-based management for uncomplicated SAM and MAM. Additionally, studies of inpatient management of SAM comparing the WHO protocol to standard care tend to be observational without adjustment for confounding. Given the types of studies available and varying contexts for many of these studies, we complemented the systematic review with a Delphi process. The purpose of the Delphi exercise was to gather and synthesize expert opinion around the plausible impact estimates of interventions in various settings [53]. We invited both researchers and practitioners who are experts in SAM and MAM to participate and provided each expert with summary data from our systematic review, details about LiST, as well as specific instructions for the Delphi process.

The Delphi consisted of three rounds. In the first round, we asked experts to provide their best estimates of CFR and recovery rate for inpatient and community management of SAM. We also asked for CFR and recovery rate for ‘optimal management’ of MAM and asked each expert to provide his or her opinion on which components constitute optimal management of MAM.

We calculated the arithmetic mean and range for each outcome and undertook thematic analysis of the qualitative data. In the second round we provided each expert with the means and ranges of each estimate, as well as a summary of the themes for optimal management of MAM. Experts were given the opportunity to refine their point estimates and to comment on the summary paragraphs. In the third round, we requested final comments or edits on the Delphi sections presented in this paper, and asked whether the experts wished to be acknowledged in this paper.

### Results from Delphi process

We received replies from 15 participants in round 1 (83%) and 13 participants responded to round 2 (72% of total, 86% of round 1 participants). All participants provided input on what constitutes ‘optimal care’ of MAM; 13 participants contributed to the mortality and recovery rates in each round.

For inpatient treatment of complicated SAM according to the WHO protocol, the panel of experts estimate a CFR of 14% (range: 5-30%) and recovery rate of 71% (25-95%). The lower bound of the recovery rate is 25% results from one expert who expressed that a large proportion of admissions would default before recovery is reached. For community-based treatment of SAM, the CFR was estimated at 4% (range: 2-7%) and a recovery rate was estimated at 80% (range: 50-93%). The mortality rate for MAM based on optimal treatment proposed by the experts (see additional file 4) was estimated at 2% (range: 0-4%) and recovery rate was 84% (50-100%).

It should be noted that true consensus in estimating CFRs and morality rates was not achieved through this process, as a few participants did not provide a single estimate for each outcome, stating that the intervention effects varied considerably by context. There was a convergence of ideas around the general approach to managing MAM, as illustrated by the major themes described in additional file 4. Consensus was not achieved regarding whether all children with MAM (in areas of high HIV prevalence) should be screened for HIV, the relative importance of each component of the management approach, or the ideal form of food to provide (whether there are other foods that are as effective as RUSF).

## Discussion

The purpose of this review was to evaluate the effectiveness of approaches to treating SAM, both the WHO protocol for inpatient management and community-based management using RUTF, as well as the effectiveness of approaches to managing MAM. In all cases we found fewer high quality studies than expected. We were unable to conduct a pooled analysis comparing the impact of the WHO protocol vs. standard care for the treatment of SAM due to the type of studies available. We conducted meta-analyses for community-based management of SAM as well as management of MAM; however, for the MAM analysis, the data available only allowed us to pool studies comparing two food commodities. Thus, we were unable to adequately evaluate the intervention effects separate from product effectiveness. While there are limitations to both the review and Delphi process that will be discussed subsequently, the estimates generated from the literature review and subsequently vetted through the Delphi process represent the next step in modeling interventions to address SAM and MAM in LiST.

The WHO protocol for the inpatient management of SAM is substantiated through considerable evidence, based both in research and expert opinion [1,54]. Several studies have demonstrated that it is possible to attain low CFRs. However, as illustrated by the lack of high quality intervention studies, lack of adjustment for confounding variables in observational studies, and absence of key details in many publications, there is a clear need for further research to improve our understanding of how to consistently achieve low CFRs across varying resource-constrained settings.

The shift to outpatient care for the treatment of uncomplicated SAM represents a major turning point in the management of severe acute malnutrition, as is has facilitated improved coverage and lower opportunity costs to caregivers [55]. Community-based management of severe acute malnutrition is backed by a wealth of observational and programmatic data [55-57], yet we found fewer impact studies than expected. While no significant difference in mortality was found in our meta-analysis, children given RUTF were 51% more likely to achieve nutritional recovery in a timely manner, though there was substantial heterogeneity. The differences in anthropometric outcomes, while statistically significant, were small and may not be clinically significant.

It should be noted that these pooled estimates were based on two cohorts of children, both in Malawi, and thus may not be generalizable. Additionally, HIV is an important factor to consider given that the HIV prevalence rate of children with SAM in Sub-Saharan Africa is high [58]. Unfortunately we were unable to disaggregate the meta-analysis as only one trial tested for HIV. A 2009 review that included children with SAM concluded that HIV-infected children are significantly more likely to die than HIV-uninfected children, but used a broader definition of acute malnutrition [58]. Much remains unclear about how to care for HIV-uninfected children with SAM [59].

The results of our meta-analysis on community-based treatment of MAM demonstrate that RUSF is slightly more beneficial than CSB. Although statistically significant, the higher rate of weight in the RUSF group is small and may not be clinically important. Children in the RUSF group were significantly more likely to recover and less likely to be non-responders. However, these estimates contained considerable levels of heterogeneity, both in terms of study design and in terms of intervention quality, which is poorly captured by most studies. Furthermore, several individual studies that we were unable to pool in our meta-analysis report modest or no statistically significant difference in key nutritional outcomes when comparing products [60-62]. There are several dozen ongoing or planned studies focused on demonstrating efficacy or effectiveness between or among a range of possible food products and nutrient supplements in the context of the management of MAM, most of which will have reports in the upcoming few years (personal communication CMAM Forum, 2012).

There are several limitations of this analysis. As some of the participants in our Delphi process indicated, outcomes of treatment programs are highly context specific and depend on background rates of HIV, seasonal fluctuations in food availability, and many other context-specific variables. Additionally, the outcomes of the programs depend not only on the products used, but the general quality of the program design and implementation, as has been noted by several researchers [16,63-65]. Despite the importance of context, intervention quality, and the linking of inpatient and outpatient treatment programs along with preventive strategies, it was not possible to undertake a disaggregated analysis by context, due to the limited number of trials available, the lack of detail given on the interventions and analysis in many studies, and the requirement for a single effect estimate in LiST.

Further to the issues inherent in the analysis, there are issues with individual studies that warrant discussion. The diets given to children were often not described in detail, and the amounts of CSB given to the comparison group varied, sometimes including enough to share with family members. Thus dietary intake of study participants is not clear in all cases. Furthermore, all but one of the studies in the meta-analysis were conducted in Africa, with a bias towards Malawi (see additional file 2), thus limiting the generalizability of the results. Additionally, all studies passively recruited participants who were brought to treatment facilities. This may introduce bias if there are systematic differences between caregivers who are more likely, and those who are less likely, to bring their children to facilities for treatment.

## Directions for future research

Our review was unable to utilize a substantial proportion of studies due to inconsistencies in admission criteria, variability in the definition of acute malnutrition (including the use of weight-for-age to assess nutritional status), and irregularities in how data is reported. In order to strengthen our understanding of the effectiveness of interventions, through the use of meta-analysis, there should be standard case definitions and reporting of outcomes at standardized time intervals. Admission criteria should be based on the WHO definition of acute malnutrition, or children meeting these criteria should be presented in a disaggregated analysis.

Further high quality impact studies of approaches to managing SAM and MAM are needed. Particularly studies that reflect a broader range of settings where these conditions are prevalent, including a range of geographic locations and areas with different disease prevalence (i.e. HIV). Though this area of research can present challenges for intervention studies, there are study design options and data analysis techniques that allow for high quality research. Where randomized controlled trials are not feasible, another option would be to employ a stepped-wedge design for research on community-based management of SAM or MAM.

Our meta-analysis was constrained with respect to the types of outcomes we were able to pool. Length of stay, relapse (requiring re-admission to the hospital), default rate, sustained recovery and cost-effectiveness were not routinely measured, but are essential factors to consider in program planning. Furthermore, all but one of the studies included in this review follow children for a relatively short period of time, providing little insight into long-term effects. A recent follow-up study by Chang et al. [66] found significant differences in sustained recovery over 12-months of follow-up, depending on the treatment given. Of all children successfully treated for MAM, sustained recovery was significantly more likely in those treated with soy/whey RUSF compared to those treated with either soy RUSF or CSB++; however, the authors concluded that all children in the study remained vulnerable. More follow-up studies are needed to illuminate long-term effects on developmental outcomes, stunting, and the transition back to a home diet. Standardized follow-up intervals over a longer time period, and reporting on a wider range of outcomes would allow for higher quality meta-analyses and a more robust understanding the intervention effects.

Similarly, trials are needed to compare different approaches for the management of MAM that consider local context, as a one-size-fits all approach is not appropriate [67]. While food supplementation is necessary in humanitarian emergencies and chronic food insecurity, acute malnutrition is not confined to situations of conflict or famine [68]. In relatively more stable situations, further research is needed on preventive approaches that address upstream determinants of acute malnutrition, illustrated by the range of ideas brought forth in the Delphi exercise (see additional file 4).

As the body of literature grows, it will also be important to disaggregate meta-analyses according to context. Therefore, greater geographic representation is needed, as are studies designed to explore the impact of factors that likely affect the individuals’ treatment outcomes, such as HIV status and household food insecurity, as well as studies that are designed to tease out the elements of successful programs, beyond the choice of commodity.

## Conclusions

The paradigm shift towards community-based treatment of SAM has transformed the approach to treating acute malnutrition. Community-based treatment is backed by substantive programmatic evidence; however, there are clear gaps in the availability of well-designed studies evaluating the effectiveness of interventions to manage SAM and MAM in a range of contexts. Thus, establishing effect estimates for LiST proved challenging. The meta-analysis demonstrates some positive effects of the use of RUTF in comparison to CSB for the treatment of SAM or MAM in the community; yet, the effects were generally small and several outcomes had substantial heterogeneity. Meanwhile, the results of the Delphi indicate that the use of standardized protocols for treating complicated SAM, uncomplicated SAM, and MAM, should lead to low mortality and high recovery rates. To close the gap between research and practice, further studies are needed that compare approaches to managing SAM and MAM, taking local context into consideration.

## Authors’ contributions

LML and ZAB conceptualized the review and analysis. LML led the systematic review and Delphi process and wrote the manuscript with substantial inputs from PW and ZAB. KW was involved in abstraction, analysis, Delphi and writing the first manuscript draft. PW and TA significantly contributed throughout the stages of the review and manuscript preparation.

## Competing interests

The authors declare that they have no competing interests.

## Supplementary Material

Additional file 1 Search Strategy Click here for file

Additional file 2 Forest plots for all meta-analysis included in review Click here for file

Additional file 3 Data abstraction and quality assessment table Click here for file

Additional file 4 **Key themes emerging from the Delphi process for the optimal management of moderate acute malnutrition** MAM: moderate acute malnutrition HIV: human immunodeficiency virus TB: tuberculosis Click here for file

## Declarations

The publication costs for this supplement were funded by a grant from the Bill & Melinda Gates Foundation to the US Fund for UNICEF (grant 43386 to "Promote evidence-based decision making in designing maternal, neonatal, and child health interventions in low- and middle-income countries”). The Supplement Editor is the principal investigator and lead in the development of the Lives Saved Tool (LiST), supported by grant 43386. He declares that he has no competing interests.

This article has been published as part of *BMC Public Health* Volume 13 Supplement 3, 2013: The Lives Saved Tool in 2013: new capabilities and applications. The full contents of the supplement are available online at http://www.biomedcentral.com/bmcpublichealth/supplements/13/S3.
