# Supplementary material for: Treatment of severe and moderate acute malnutrition in low- and middle-income settings: a systematic review, meta-analysis and Delphi process
Source: BMC Public Health. 2013 Sep 17;13(Suppl 3):S23. doi: 10.1186/1471-2458-13-S3-S23 (PMC3847503; doi:10.1186/1471-2458-13-S3-S23)
Supplement: Additional file 1 — Search Strategy [file 1471-2458-13-S3-S23-S1.docx]

**Medline:**

1 nutrition disorders/ or child nutrition disorders/ or infant nutrition disorders/ or malnutrition/ or deficiency diseases/ or exp protein deficiency/ or starvation/ (53293)

2 (nutrition adj2 disorder*).mp. (23320)

3 ((nutrition* or protein or food or nutrient* or nutritive* or alimentary or diet*) adj2 (deficien* or insufficien* or deficit*)).mp. (24857)

4 (malnutrition* or "deficiency disease*" or undernutrition* or underfeed* or undernourish* or undernutrition* or marasmus or kwashiorkor* or famine* or starvation* or wasting or wasted or GAM or MAM or SAM or malnourish*).mp. (87824)

5 exp Wasting Syndrome/ (1304)

6 1 or 2 or 3 or 4 or 5 (120332)

7 food, formulated/ or food, fortified/ (12206)

8 dietary fats/ or dietary proteins/ or milk proteins/ or dietary supplements/ (98298)

9 ((fortif* or enrich* or formulat* or supplement* or lipid*) adj2 (food* or spread* or diet*)).mp. (61358)

10 (F-75 or F-100 or F75 or F100 or feeds or "therapeutic feed*" or "supplement* feed*" or CMAM or CTC or RUF or RUTF or "ready to use therapeutic food*" or "ready to use food*" or "ready to use supplement* food*" or RUSF or plumpy* or eeZeeRUSF or FBF or "fortified blended flour*" or supercereal* or "corn soy* blend*" or "wheat soy* blend*" or "rice milk blend*" or "milk rice blend*" or "pea wheat blend*" or "cereal pulse blend" or MRB or CSB or "lipid-based nutrient supplement*" or Nutributter or LNS).mp. (13522)

11 ("alimentary fat*" or "fat consumption" or "fat feed*" or "fat ingestion" or "fatty acid intake" or "lipid intake" or "fat nutrition" or "food protein*" or "protein consumption" or "protein feed*" or "protein food" or "protein nutrition" or "protein intake" or "whey protein*").mp. (12604)

12 (treat* or therap* or manag* or supplement* or rehabilitat*).mp. (5100345)

13 or/7-12 (5173624)

14 6 and 13 (41545)

15 limit 14 to ("newborn infant (birth to 1 month)" or "infant (1 to 23 months)" or "preschool child (2 to 5 years)") (6947)

16 (infan* or newborn* or new-born* or neonat* or baby or babies or child* or kid or kids or toddler* or boy* or girl* or p?ediatric* or preschooler* or pre-schooler*).mp. (2365885)

17 14 and 16 (11478)

18 15 or 17 (11478)

19 ("clinical trial, all" or clinical trial).pt. or clinical trials as topic/ (566917)

20 clinical trial, phase i.pt. or clinical trials, phase i as topic/ (16379)

21 clinical trial, phase ii.pt. or clinical trials, phase ii as topic/ (25381)

22 clinical trial, phase iii.pt. or clinical trials, phase iii as topic/ (12813)

23 clinical trial, phase iv.pt. or clinical trials, phase iv as topic/ (945)

24 controlled clinical trial.pt. or controlled clinical trials as topic/ (90046)

25 meta-analysis.pt. or meta-analysis as topic/ (48389)

26 multicenter study.pt. or multicenter studies as topic/ (162790)

27 randomized controlled trial.pt. or randomized controlled trials as topic/ (417508)

28 exp case-control studies/ or exp cohort studies/ or cross-sectional studies/ (1453803)

29 (cohort* or case-control* or cross-sectional* or RCT or multicentre or multicenter).mp. (751010)

30 ((concurrent or incidence or case-comparison or case-referrent or case-compeer or case-base or prevalence) adj2 (study or studies)).mp. (13389)

31 (placebo* or random*).mp. (798874)

32 ((singl* or doubl* or trebl* or tripl*) adj2 (mask* or blind*)).mp. (161280)

33 (latin adj2 square).mp. (3056)

34 comparative study/ or evaluation studies/ (1738664)

35 Evaluation Studies as Topic/ (119835)

36 ((longitudinal or prospective or retrospective or intervention or comparative or evaluation or operational) adj2 (study or studies)).mp. (2556536)

37 (metaanalys* or meta-analys* or review*).mp. (2260540)

38 ((clinical or control* or field or cluster*) adj2 trial*).mp. (911839)

39 cross-over studies/ (30682)

40 ((crossover or cross-over) adj2 (study or studies or trial*)).mp. (42914)

41 (interrupted adj2 time adj2 series).mp. (728)

42 (control* adj2 before adj2 after adj2 (study or studies)).mp. (334)

43 or/19-42 (5742365)

44 18 and 43 (5878)

**Embase:**

1 malnutrition/ or nutritional disorder/ or exp protein deficiency/ (67590)

2 starvation/ (13734)

3 wasting syndrome/ (2682)

4 (nutrition adj2 disorder*).mp. (1654)

5 ((nutrition* or protein or food or nutrient* or nutritive* or alimentary or diet*) adj2 (deficien* or insufficien* or deficit*)).mp. (46026)

6 (malnutrition* or "deficiency disease*" or undernutrition* or underfeed* or undernourish* or undernutrition* or marasmus or kwashiorkor* or famine* or starvation* or wasting or wasted or GAM or MAM or SAM or malnourish*).mp. (110887)

7 1 or 2 or 3 or 4 or 5 or 6 (160134)

8 diet supplementation/ (55953)

9 fat intake/ or protein intake/ (60925)

10 milk protein/ (7409)

11 ((fortif* or enrich* or formulat* or supplement* or lipid*) adj2 (food* or spread* or diet*)).mp. (126403)

12 (F-75 or F-100 or F75 or F100 or feeds or "therapeutic feed*" or "supplement* feed*" or CMAM or CTC or RUF or RUTF or "ready to use therapeutic food*" or "ready to use food*" or "ready to use supplement* food*" or RUSF or plumpy* or eeZeeRUSF or FBF or "fortified blended flour*" or supercereal* or "corn soy* blend*" or "wheat soy* blend*" or "rice milk blend*" or "milk rice blend*" or "pea wheat blend*" or "cereal pulse blend" or MRB or CSB or "lipid-based nutrient supplement*" or Nutributter or LNS).mp. (19105)

13 ("alimentary fat*" or "fat consumption" or "fat feed*" or "fat ingestion" or "fatty acid intake" or "lipid intake" or "fat nutrition" or "food protein*" or "protein consumption" or "protein feed*" or "protein food" or "protein nutrition" or "protein intake" or "whey protein*").mp. (39616)

14 (treat* or therap* or manag* or supplement* or rehabilitat*).mp. (7854042)

15 or/8-14 (7940602)

16 7 and 15 (63709)

17 limit 16 to (infant <to one year> or preschool child <1 to 6 years>) (3936)

18 (infan* or newborn* or new-born* or neonat* or baby or babies or child* or kid or kids or toddler* or boy* or girl* or p?ediatric* or preschooler* or pre-schooler*).mp. (2709130)

19 16 and 18 (15931)

20 17 or 19 (15931)

21 exp case control study/ or exp clinical trial/ or exp "clinical trial (topic)"/ or intervention study/ or longitudinal study/ or prospective study/ (1268930)

22 cohort analysis/ or cross-sectional study/ or crossover procedure/ or latin square design/ (245620)

23 meta analysis/ or "meta analysis (topic)"/ (70484)

24 comparative study/ (685641)

25 evaluation/ (180558)

26 (cohort* or case-control* or cross-sectional* or RCT or multicentre or multicenter).mp. (748618)

27 ((concurrent or incidence or case-comparison or case-referrent or case-compeer or case-base or prevalence) adj2 (study or studies)).mp. (19024)

28 (placebo* or random*).mp. (1047910)

29 ((singl* or doubl* or trebl* or tripl*) adj2 (mask* or blind*)).mp. (196419)

30 (latin adj2 square).mp. (3436)

31 ((longitudinal or prospective or retrospective or intervention or comparative or evaluation or operational) adj2 (study or studies)).mp. (1464931)

32 (metaanalys* or meta-analys* or review*).mp. (2830671)

33 ((clinical or control* or field or cluster*) adj2 trial*).mp. (1138494)

34 ((crossover or cross-over) adj2 (study or studies or trial*)).mp. (33571)

35 (interrupted adj2 time adj2 series).mp. (909)

36 (control* adj2 before adj2 after adj2 (study or studies)).mp. (409)

37 or/21-36 (5673531)

38 20 and 37 (6840)
