# Supplementary material for: Treatment of severe and moderate acute malnutrition in low- and middle-income settings: a systematic review, meta-analysis and Delphi process
Source: BMC Public Health. 2013 Sep 17;13(Suppl 3):S23. doi: 10.1186/1471-2458-13-S3-S23 (PMC3847503; doi:10.1186/1471-2458-13-S3-S23)
Supplement: Additional file 3 — Data abstraction and quality assessment table [file 1471-2458-13-S3-S23-S3.docx]

Table of Contents:

1. **Moderate Acute Malnutrition: Supplementary Feeding**
   1. Mortality
   2. Non-Response Rate
   3. Recovery Rate
   4. Rate of Height Gain
   5. Rate of MUAC Gain
   6. Rate of Weight Gain
   7. Weight-for-height Z-score at completion of intervention or discharge
2. **Severe Acute Malnutrition: Therapeutic Feeding**
   1. Mortality
   2. Rate of Height Gain
   3. Recovery Rate
   4. Rate of MUAC Gain
   5. Rate of Weight Gain
      1. In RUTF vs. CSB
      2. Imported vs. Local RUTF
3. **Severe Acute Malnutrition: Inpatient vs. Ambulatory Care**
   1. Mortality

**1. Moderate Acute Malnutrition: Supplementary Feeding**

**1.1 Mortality:**


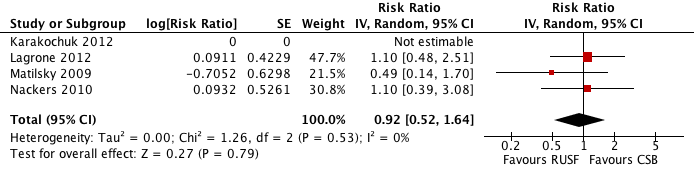


**1.2 Non-Response Rate:**

**
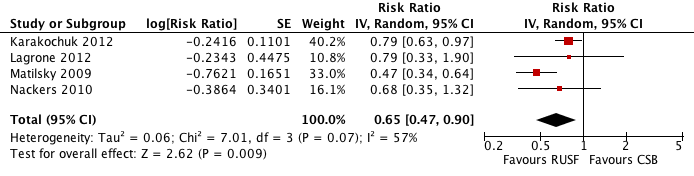
**

**1.3 Recovery Rate:**

**
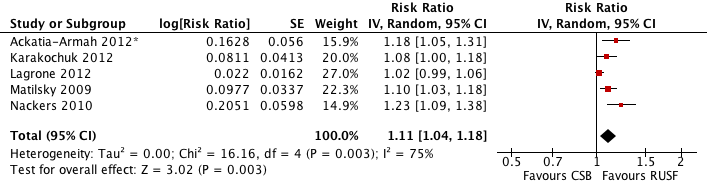
**

**1.4 Rate of Height Gain:**

**
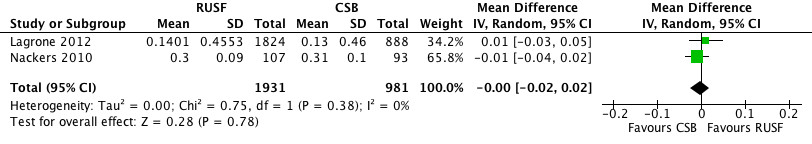
**

**1.5 Rate of MUAC Gain:**

**
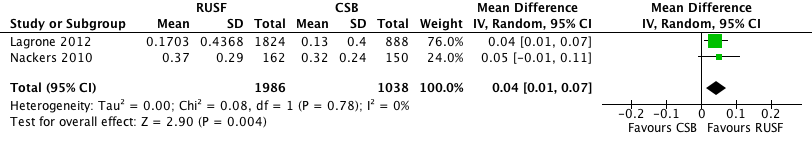
**

**1.6 Rate of Weight Gain:**

**
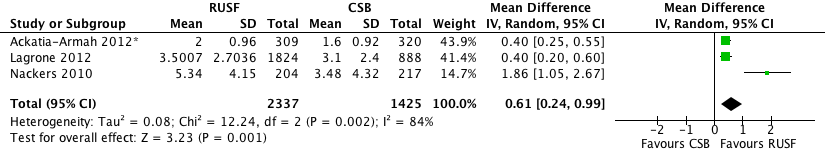
**

**1.7 Change in Weight-for-Height Z-Score At Completion or Discharge:**

**
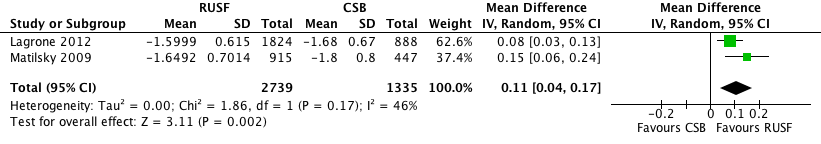
**

**2. Severe Acute Malnutrition: Therapeutic Feeding**

**2.1 Mortality:**

**
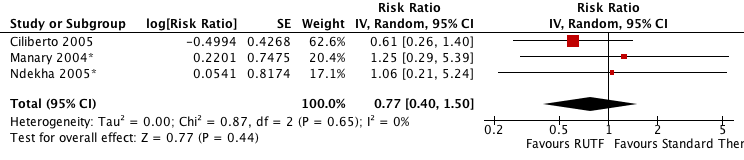
**

**2.2 Rate of Height Gain:**

**
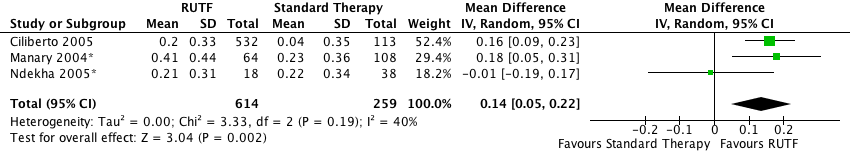
**

**2.3**  **Recovery Rate:**

**
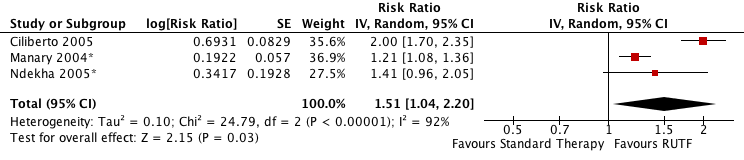
**

**2.4 Rate of MUAC Gain:**

**
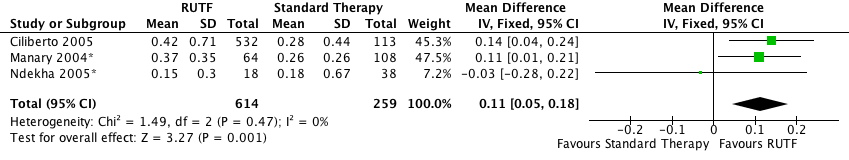
**

**2.5 Rate of Weight Gain:**

**2.5.1 In RUTF vs. CSB**

**
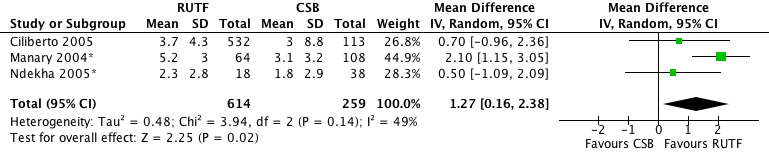
**

**2.5.2 Imported vs. Local RUTF**

**
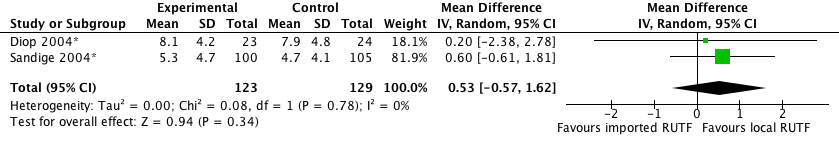
**

**3. Types of Care:**

**3.1 Mortality:**

**
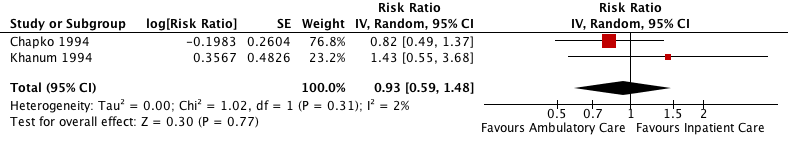
**
