# Supplementary material for: Treatment of severe and moderate acute malnutrition in low- and middle-income settings: a systematic review, meta-analysis and Delphi process
Source: BMC Public Health. 2013 Sep 17;13(Suppl 3):S23. doi: 10.1186/1471-2458-13-S3-S23 (PMC3847503; doi:10.1186/1471-2458-13-S3-S23)
Supplement: Additional file 4 — Key themes emerging from the Delphi process for the optimal management of moderate acute malnutrition MAM: moderate acute malnutrition HIV: human immunodeficiency virus TB: tuberculosis [file 1471-2458-13-S3-S23-S4.docx]

**Additional file 4 – Key themes emerging from the Delphi process for the optimal management of moderate acute malnutrition**

- Promote the intake of an energy-dense diet that has the right balance of high-quality protein, essential fatty acids and the micronutrients required to promote lean-tissue growth. This may be achieved through careful planning of the diet based on home foods accompanied by an age-appropriate multiple micronutrient supplements or through provision of a food specially formulated to meet these requirements such as an RUSF or a fortified cereal blend porridge containing milk powder. Additional micronutrients should be provided based on a case-by-case basis, if a deficiency is detected that requires treatment.
- Provide supplemental food when adequate diet quality cannot be achieved through home-based foods, such as in situations of food insecurity and population displacement. In these settings, a household food ration should also be provided to discourage sharing.
- Provide medical treatment for any condition detected; take measures to prevent disease, including updating vaccines, supplying bed nets, deworming children as well promoting WASH (water, hygiene, sanitation); integrate vertical programs into the three-tier health system; treatment for moderate acute malnutrition should be linked to treatment and referral systems for severe acute malnutrition and to preventive strategies; staff should be well-trained at all levels.
- Understand possible underlying reasons for MAM, and address accordingly, this may include screening for HIV and TB or developing psychosocial programs to address inadequate care practices.
- Develop community-specific nutrition and illness-prevention education programs, with content tailored to the local context; adequate time should be allotted to this education; staff should be carefully trained and motivated to counsel.
- Follow-up and reassess children frequently; make efforts to locate all defaulters.
- Address the upstream determinants of malnutrition. Examples provided by the experts include social protection programs, agricultural diversification, as well as applying a rights-based and gender-based approach).
- Invest in local technology and capacity to produce high quality foods (supplemental and otherwise).
- Engage and empower communities in the care of malnourished children; a respectful, caring environment should be cultivated between caregivers, health care workers and researchers.
